# Enteroscopic direct drainage for submucosal intestinal juice leakage due to afferent loop syndrome in Roux-en-Y anatomy

**DOI:** 10.1055/a-2652-3508

**Published:** 2025-08-08

**Authors:** Mitsuru Okuno, Tsuyoshi Mukai, Fumiya Kataoka, Hiroshi Araki, Eiichi Tomita, Hisataka Moriwaki, Masahito Shimizu

**Affiliations:** 1 73505 Department of Gastroenterology, Matsunami General Hospital, Gifu, Japan; 2 73505 First Department of Internal Medicine, Gifu University Hospital, Gifu, Japan

We report a case of intestinal fluid accumulation secondary to afferent loop syndrome, successfully treated via enteroscopic submucosal drainage.

A 66-year-old man with a history of gastric resection and Roux-en-Y reconstruction for gastric cancer 6 years earlier presented with fever. Computed tomography (CT) revealed liver metastasis invading the bile duct near the duodenal end loop ( Fig. 1 ). Enteroscopy confirmed tumor invasion near the end loop of the duodenum, with no additional intestinal abnormalities. Suspecting cholangitis, bilateral biliary plastic stent (PS) drainage was performed using an enteroscope (EI-580BT; Fujifilm, Tokyo, Japan), and the patient was discharged after clinical improvement.

**Fig. 1:**
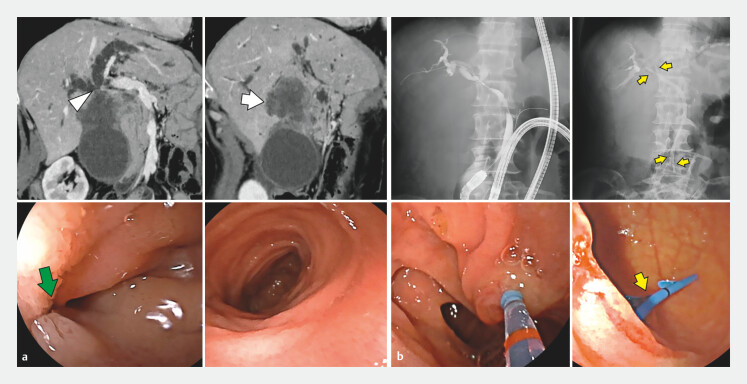
**a** Computed tomography reveals a liver metastasis (arrow) with invasion to the bile duct (arrowhead) in a patient with Roux-en-Y reconstruction. Enteroscopy shows tumor invasion near the duodenal end loop (green arrow) without other intestinal abnormalities. **b** Bilateral biliary plastic stent drainage (yellow arrow) was performed under enteroscopic guidance.

Three weeks later, he developed abdominal pain. CT revealed a distended end loop and submucosal expansion in the horizontal part of the duodenum ( Fig. 2 ), suggesting leakage of accumulated intestinal fluid from the end-loop cavity into the duodenal submucosal space. Endoscopic ultrasound failed to access the site. Therefore, an enteroscope was used, and the tensed mucosa was punctured with a precut needle knife, with the elasticity of the mucosal surface assessed prior to entry. A 6-mm dilation balloon was used to expand the puncture tract, and both 7-Fr PS and 6-Fr drainage tubes were placed in the fluid collection cavity. One week later, fluoroscopy confirmed collapse of the fluid collection, and the 6-Fr tube was removed ( Video 1 ).

**Fig. 2:**
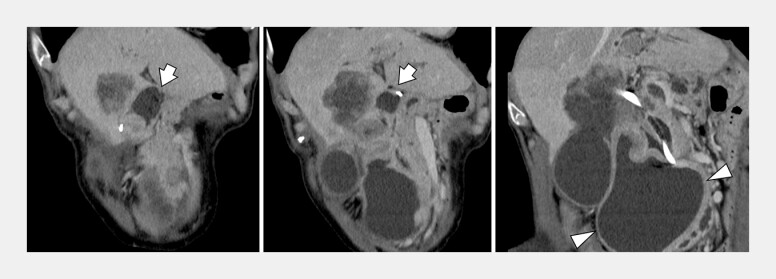
Computed tomography shows a tensed end loop (arrow) and expansion of the submucosal space in the horizontal part of the duodenum (arrowhead).

Massive submucosal fluid collection in the duodenum was drained using a precut needle knife under enteroscopy in a case of afferent loop syndrome with Roux-en-Y anatomy.Video 1

Although endoscopic ultrasound-guided gastroenterostomy is a safe option for afferent loop syndrome, as it allows the evaluation of blood vessels and needle access to the afferent limb 1 2 3 , access was limited in this case due to Roux-en-Y anatomy. Direct enteroscopic puncture poses a perforation risk but was safely performed with mucosal elasticity assessment. Fluid accumulated after drainage tube removal, though the patient remained asymptomatic, with partial drainage via the remaining 7-Fr PS ( Fig. 3 ).

**Fig. 3:**
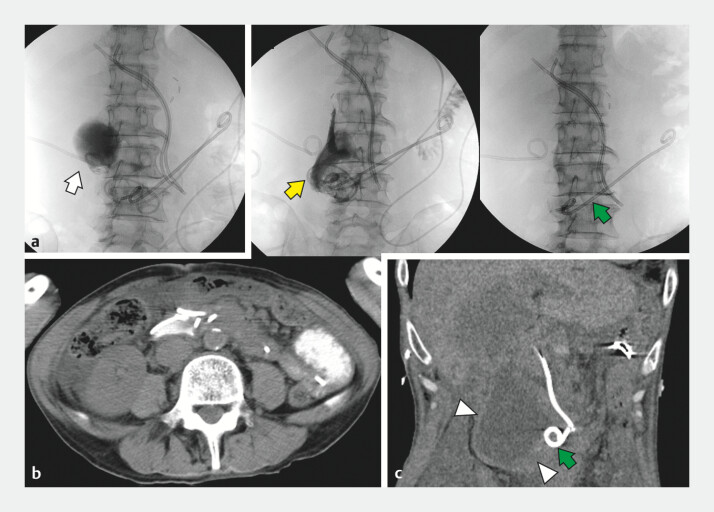
**a** PTGBD (arrow) is performed. No communication was observed between the gallbladder and the duodenal submucosal cavity. **b** Fluoroscopy and computed tomography demonstrate collapse of the fluid collection. After which, the 6-Fr drainage tube (yellow arrow) was removed. **c** Following drainage tube removal, fluid retention reappeared (arrowhead), although the patient remained asymptomatic. It was considered that intestinal juice continued to drain partially through the remaining 7-Fr plastic stent (green arrow). PTGBD: percutaneous transhepatic gallbladder drainage.

Endoscopy_UCTN_Code_TTT_1AP_2AD

## Notes

Endoscopy E-Videos https://eref.thieme.de/e-videos
*E-Videos* is an open access online section of the journal *Endoscopy* , reporting on interesting cases and new techniques in gastroenterological endoscopy. All papers include a high-quality video and are published with a Creative Commons CC-BY license. Endoscopy E-Videos qualify for HINARI discounts and waivers and eligibility is automatically checked during the submission process. We grant 100% waivers to articles whose corresponding authors are based in Group A countries and 50% waivers to those who are based in Group B countries as classified by Research4Life (see: https://www.research4life.org/access/eligibility/ ). This section has its own submission website at https://mc.manuscriptcentral.com/e-videos .
